# Snack consumption frequency of children and adults in the Vaal region of Gauteng, South Africa

**DOI:** 10.4102/hsag.v28i0.2181

**Published:** 2023-11-15

**Authors:** Temitope E. Ibiyemi, Wilna H. Oldewage-Theron

**Affiliations:** 1 Department of Nutritional Sciences, Faculty of Human Sciences, Texas Tech University, Lubbock, United States; 2 Department of Sustainable Food Systems and Development, Faculty of Natural and Agricultural Science, University of the Free State, Bloemfontein, South Africa

**Keywords:** snacks, snacking preferences, snack consumption, children, adults, low socioeconomic status

## Abstract

**Background:**

Energy-dense, nutrient-poor snacks are associated with an increased risk of non-communicable diseases (NCDs) and the double burden of malnutrition, especially among poor communities.

**Aim:**

To determine and compare the snacking preferences and consumption frequency of children and adults from a low SES community in South Africa.

**Setting:**

A preschool, primary school, and an elderly centre in Gauteng.

**Methods:**

A cross-sectional study with a convenience sample of 90 children (3–8 years) and 100 adults (≥ 18 years) to assess snack consumption frequency and preferences of children and adults by means of a snack survey. Mann-Whitney U test was used to determine the differences in preferences and snack consumption frequency of children and adults.

**Results:**

The most preferred and consumed snacks included fruits, potato chips, corn chips, sweets, and cookies. Children frequently ate more potato chips (*p* < 0.001), corn chips (*p* < 0.001), cheese curls (*p* < 0.001), and muffins (*p* = 0.024) than adults. In contrast, adults frequently consumed more peanuts or nuts (*p* = 0.024), savoury biscuits (*p* = 0.048) and biltong (*p* < 0.001) than children.

**Conclusion:**

Apart from fruits, the most preferred and frequently consumed snacks by the sample were highly processed snacks, which are low in fibre and high in added sugars, saturated fat, and sodium.

**Contributions:**

Findings from this study highlight current snack trends and can guide future nutrition education interventions on healthy snacking and in developing nutritious snacks for the South African community

## Introduction

Over the last decades, snack consumption has been rising in South Africa (Hassanally, Naicker & Singh 2020; Ronquest-Ross, Vink & Sigge 2015). This increase in snack consumption is of public concern because of the connection to the double burden of malnutrition (DBM) – the coexistence of undernutrition and overweight or obesity in the same population, community, household or person (Mkhize, Napier & Oldewage-Theron 2013; Moyo, Egal & Oldewage-Theron 2022; Popkin, Corvalan & Grummer-Strawn 2020). In a 2020 Lancet series, the consumption of cheap snacks and beverages was identified as the leading risk factor for DBM, particularly among low- and middle-income countries (LMICs) such as South Africa (Popkin et al. 2020; Wells et al. 2020).

Interestingly, South Africa is facing the DBM. The latest South African Health and Demographic Survey (SADHS) 2016 reported that although undernutrition was still prevalent in 27% of children under 5 years (U5) being stunted – with 10% severely stunted – overweight was also prevalent in 13% of U5 children (National Department of Health [NDoH], StatsSA & SAMRC 2019; StatsSA 2017). Among older children and adolescents (5–19 years), 29.4% of girls and 20.2% of boys are overweight, while 3.4% of girls and 6.0% of boys are underweight. The prevalence of obesity among older children and adolescents is 12.8% among girls and 9.8% among boys. Likewise, the prevalence of obesity in South African women is almost four times that in men, while underweight in men is three times higher than in women (McHiza et al. 2019). There is thus a trend that the prevalence of being overweight is increasing with age in the country.

South Africa has been experiencing a nutritional transition over the last two decades (Vorster, Kruger & Margetts 2011; Wrottesley et al. 2019; Zingoni et al. 2009). In particular, this transition has been characterised by increased consumption of sugary drinks, sweets, savoury snacks, and ultra processed and packaged snack foods that are lower in fibre and high in salt, sugar and saturated fats (NDoH 2022). Although some snacks can provide essential nutrients, majority of the consumed snacks in South Africa are nutrient-poor and energy-dense. These popular snacks in the South African market, such as chips, puffs, corn bites, toffees and chocolates, are high in added sugars and sodium, which is inconsistent with healthy dietary recommendations to combat obesity and undernourishment (Eagle 2019; Pries, Filteau & Ferguson 2019). In 2017, maize, potato chips and puffs dominated the South African snack industry with 40%, 20% and 10% of the market sector, respectively; whereas jellies and gums dominated the sugar confectionery industry (Eagle 2019). The South African savoury snacks market is projected to grow by 31.6% from 2019 to 2026 (Statista 2021). Such snacks, high in added sugars, saturated fat and sodium, are usually cheaper than nutritious foods and thus widely consumed by children and adults. A review of food consumption patterns from 1994 to 2012 in South Africa indicates an increase in sugar confectionery consumption of 16.7%. Total biscuit (cookie) consumption increased by 57.1%, while sweet and savoury snacks increased by 53.3% (Ronquest-Ross et al. 2015). In 2016, SADHS reported that 41.0% of children consumed salty snacks while 35.0% consumed sugary snacks (NDoH et al. 2019).

Taste, affordability and availability are the most common factors leading to the continuous increase in snack consumption in South Africa (Govender et al. 2018; Wrottesley et al. 2020). The healthier snacks are generally more expensive than most available snacks (Govender et al. 2018; Herforth et al. 2020). This high cost, coupled with the country’s low socioeconomic status (SES), makes inexpensive, energy-dense, nutrient-poor snacks the preferred choice among the South African population (Hassanally et al. 2020; Steyn, Labadarios & Nel 2011).

With the onset of COVID-19, the affordability and availability of foods have been affected (Litton & Beavers 2021). In South Africa, snack sales and money spent on snacks have risen. An Institute of Public Sector Opinion Survey (IPSOS) consumption and consumer survey reported an increase of 43% in snack sales by October 2021 compared to the previous year (IPSOS South Africa 2021). Given the rise in snack sales and the DBM in South Africa, the need for a snack consumption survey that can guide future nutrition education interventions for consumers on healthy snacking and inform the development of nutritious snack options has become increasingly apparent.

One of the drawbacks of snack consumption frequency studies is that there is no generalised or standardised definition of snacks (Hess, Jonnalagadda & Slavin 2016; Potter, Vlassopoulos & Lehmann 2018). As a result, consuming nutritious food items such as fruits and nuts consumed as snacks can be seen as snacking with a negative connotation. While previous studies have reported snack preferences and choices in South Africa, this study aims to contribute to prior knowledge by utilising more than 20 specific examples of common snacks consumed in the country. This knowledge of specific snack consumption frequency and preferences will serve as a guide in developing nutrient-dense snacks that can replace the energy-dense, nutrient-poor snacks currently available in the South African market, inform nutrition education programmes on healthy snacking and promote healthy snacking behaviours among communities of low SES. Hence, the objective of the study was to determine and compare the snacking preferences and frequency of children and adults from a low SES community in South Africa. Children and adults have distinct social, physiological and environmental factors that can influence snack preferences and consumption (Almoraie et al. 2021; Gubbels 2020; Scaglioni et al. 2018). By assessing and comparing snack preferences and consumption frequency of commonly consumed snacks between children and adults, this study will provide a comprehensive understanding of snack behaviours across the two age groups.

## Research methods and design

### Study design and participants

This study was conducted in the Vaal region of South Africa — located approximately 70 km south of Johannesburg in the Gauteng province. With about 800 000 people, Vaal is a densely populated region in the province of Gauteng (Oldewage-Theron, Kruger & Egal 2014). Gauteng is the country’s financial hub and the most populated province, with 15.5 million people, about 26% of the country’s total population (Statistics South Africa 2020).

Participants (children 3–8 years and adults ≥18 years) were recruited for this study via a convenient sampling method and completed a snack consumption frequency and preference survey. The investigators visited a preschool, a primary school and an elderly home in the Vaal region and obtained permission to undertake the study from the school principals and coordinator of the elderly home. Children were recruited from a preschool and a primary school in the region, whereas adults were recruited in various ways. Firstly, parents of preschool and primary school students were recruited. Secondly, attendees of an elderly care centre were recruited as adult respondents. All interested adult participants and parents of children were approached, and those who gave consent or parental consent for their children completed the questionnaire between 15 June 2021 and 23 June 2021.

### Ethical consideration

Ethics approval for this study was sought from the Institutional Review Board (IRB) at the University of the Free State (UFS) (ethical clearance number – UFS-HSD2021/0821/22). The snack preference questionnaire was developed from a previously used and validated snack pattern questionnaire in the Vaal region (Du Plessis 2009). All questionnaires were completed by participants, while parents of children ≤ 5 years completed the questionnaire on their behalf. Survey respondents were asked to indicate how much they liked the typical snacks consumed in South Africa, how often they are consumed and how much is typically spent on snacks per week. For this study, snacks were defined as foods consumed between regular meals.

### Data analyses

The completed paper questionnaires were entered into a Microsoft Excel 2016 spreadsheet. The Statistical Package for Social Sciences (SPSS) software version 28 (IBM SPSS Version 28.0.) was used to analyse the data collected. Descriptive statistics were calculated as percentages and presented as numbers and percentages (*n*[%]). The Mann-Whitney U non-parametric test was utilised to determine the difference between children’s and adults’ consumption and liking of different snacks, with a *p*-value < 0.05 considered significant. Spearman’s rank correlation was done to assess the association between snack consumption frequency and liking of the snacks. The definitions of the correlation values are 0–0.19, regarded as ‘very weak’; 0.2–0.39 as ‘weak’; 0.40–0.59 as ‘moderate’; 0.6–0.79 as ‘strong’ and 0.80–1 as ‘very strong’. To calculate the mean score of snack consumption frequency, ‘daily’, ‘weekly’, ‘weekend treats’, ‘seldom’ and ‘never’ were given a score of 7, 1, 1, 0.5 and 0, respectively. Whereas for the liking of snacks, ‘I really like it’, ‘I like it a little’, ‘It is okay’, ‘I do not like it’, ‘I really do not like it’ and ‘I have never tasted it’ were given a score of 5, 4, 3, 2, 1 and 0, respectively.

## Results

### Study participants’ characteristics

A total of 204 completed questionnaires were retrieved. Fourteen respondents who did not include their age and completed less than 20% of the survey were removed. Resultantly, 190 respondent data — 90 children aged 3–8 years and 100 adults aged ≥ 18 years — who completed the questionnaire were analysed. Among the children, 49% of the respondents were boys, while 51% were girls. The mean age of children was 5.77 years (SD 1.77). Among adult participants, 69% were women, 31% were men and the mean age of adults was 34.96 years (SD 13.10). More than two-thirds of the children (68.2%) consumed snacks at least once a day, while 46% of adults consumed snacks at least once a day (Table 1). The majority of children (58%) and adults (54%) spent up to ZAR50 (South African Rand) on snacks weekly. More than one-third of children and adults (39.1% and 32.6%, respectively) snacked most when watching television (TV). Similarly, about one-third (32%) of adults and 18% of children snacked most when bored. Table 1 presents the complete age and gender distribution, snacking frequency and amount spent on snacks by the participants.

**TABLE 1:** Characteristics of participants.

Characteristics	Children (*N* = 90)			Adults (*N* = 100)			Total participants (*N* = 190)		
	Mean ± s.d.	*n*	%	Mean ± s.d.	*n*	%	Mean ± s.d.	*n*	%
**Age**	5.77 ± 1.81	-	-	34.96 ± 13.10	-	-	-	-	-
**Gender**									
Male	-	44	48.9	-	31	31.0	-	74	39.6
Female	-	46	51.1	-	69	69.0	-	113	60.4
**How often do you eat snack food items**									
3–4 times a day	-	13	14.8	-	5	5.0	-	18	9.6
1–2 times a day	-	47	53.4	-	41	41.0	-	87	46.5
3–4 times a week	-	17	19.3	-	21	21.0	-	38	20.3
1–2 times a week	-	8	9.1	-	16	16.0	-	24	12.8
3–4 times a month	-	3	3.4	-	9	9.0	-	12	6.4
1–2 times a month	-	0	0	-	5	5.0	-	5	2.7
Seldom	-	0	0	-	3	3.0	-	3	1.6
Never	-	0	0	-	0	0	-	0	0
**Amount spent on snacks in a week**									
ZAR < 25	-	31	34.8	-	25	24.2	-	56	29.8
ZAR 26–50	-	21	23.6	-	29	30.3	-	50	26.6
ZAR 51–75	-	14	15.7	-	12	12.1	-	26	13.8
ZAR 76–100	-	10	11.2	-	15	15.2	-	25	13.3
ZAR 101–125	-	7	7.9	-	7	7.1	-	14	7.4
ZAR 125–150	-	1	1.1	-	6	6.1	-	7	3.7
ZAR > 150	-	5	5.6	-	5	5.1	-	10	5.3
**When do you snack most?**									
Hungry	-	8	9.2	-	14	14.7	-	22	12.1
Bored	-	-	-	-	30	31.6	-	46	25.3
Stressed	-	1	1.1	-	10	10.5	-	11	6.0
Watching TV	-	34	39.1	-	31	32.6	-	65	35.7
Playing on internet or other games	-	7	8.0	-	3	3.2	-	10	5.5
Visiting with friends	-	10	11.5	-	3	3.2	-	13	7.1
Other times	-	11	12.6	-	4	4.2	-	15	8.2

ZAR, South African Rand; TV, television; s.d., standard deviation.

### Snack consumption frequency

The mean consumption frequency score shows that the five most frequently consumed snacks by children included fruits, potato chips, corn chips, sweets and cookies. The most frequently consumed snacks among adults included fruits, sweets, freshly fried chips, cookies and corn chips (Table 2). Children consumed more potato chips, corn chips, cheese curls and muffins than adults. In contrast, adults consumed more peanuts, savoury biscuits and biltong than children (Table 2). These differences between adults and children were statistically significant (*p* < 0.05).

**TABLE 2:** Snack frequency of participants reported in percentage.

Snack	Participants	Daily (%)	Weekly (%)	Weekend treats (%)	Seldom (%)	Never (%)	Mean score	*P*-value
Potato chips	Children	37.6	25.9	15.3	21.2	0	3.153	**< 0.001**
	Adults	9.1	31.3	30.3	28.3	1.0	1.394	
Corn chips	Children	34.9	31.4	14.0	15.1	4.7	2.971	**< 0.001**
	Adults	17.3	23.5	22.4	32.7	4.1	1.837	
Cheese curls	Children	6.8	19.3	17.0	42.0	14.8	1.051	**< 0.001**
	Adults	3.1	6.3	5.2	44.8	40.6	0.557	
Popcorn	Children	9.9	18.5	19.8	43.2	8.6	1.290	0.205
	Adults	6.2	14.4	19.6	48.5	11.3	1.015	
Peanuts or nuts	Children	6.2	12.3	13.6	46.9	21.0	0.926	**0.024**
	Adults	11.2	23.5	12.2	38.8	14.3	1.337	
Fruit (fresh, canned or dried)	Children	51.2	19.8	5.8	14.0	9.3	3.907	0.506
	Adults	39.8	32.7	12.2	9.2	6.1	3.287	
Raisins	Children	2.5	8.8	10.0	38.8	40.0	0.556	0.271
	Adults	2.1	6.3	13.7	49.5	28.4	0.595	
Sweets	Children	17.6	21.2	36.5	18.8	5.9	1.906	0.976
	Adults	28.1	18.8	15.6	26.0	11.5	2.443	
Snack bars	Children	4.8	10.8	33.7	36.1	14.5	0.964	0.620
	Adults	6.4	18.1	14.9	43.6	17.0	0.995	
Chocolates	Children	3.6	19.0	41.7	28.6	7.1	1.000	0.706
	Adults	8.2	28.6	20.4	31.6	11.2	1.219	
Rusk	Children	6.1	12.2	4.9	28.0	48.8	0.738	0.686
	Adults	2.1	10.6	8.5	37.2	41.5	0.527	
Cookies	Children	12.9	37.6	28.2	16.5	4.7	1.647	0.865
	Adults	17.2	33.3	20.2	25.3	4.0	1.864	
Biltong or dried wors	Children	0	3.6	14.5	42.2	39.8	0.392	**< 0.001**
	Adults	7.2	16.5	18.6	40.2	17.5	1.057	
Savoury biscuits	Children	4.9	6.1	9.8	24.4	54.9	0.622	**0.048**
	Adults	4.2	7.4	9.5	44.2	34.7	0.684	
Cakes	Children	4.7	14.0	29.1	48.8	3.5	1.000	0.403
	Adults	4.1	14.3	25.5	45.9	10.2	0.913	
Tarts	Children	0	3.8	20.5	37.2	38.5	0.429	0.624
	Adults	1.0	2.1	15.5	42.3	39.2	0.459	
Scones or muffins	Children	13.1	25.0	34.5	25.0	2.4	1.637	**0.024**
	Adults	6.2	24.7	22.7	42.3	4.1	1.119	
Ice cream	Children	2.4	10.7	42.9	36.9	7.1	0.887	0.768
	Adults	4.2	11.5	35.4	41.7	7.3	0.969	
Condensed milk	Children	4.8	4.8	3.6	21.4	65.5	0.524	0.176
	Adults	1.0	5.1	2.0	40.4	51.5	0.343	
Chips (fresh fried)	Children	7.2	39.8	34.9	16.9	1.2	1.337	0.201
	Adults	16.5	41.2	21.6	16.5	4.1	1.866	
High-protein snacks	Children	13.8	30.0	8.8	25.0	22.5	1.475	0.783
	Adults	12.4	28.9	14.4	27.8	16.5	1.438	

Note: *P*-value < 0.05 is significant.

More than one-third of children reported they consumed corn chips, potato chips and fruits daily. In comparison, 17% of adults consumed freshly fried chips, potato chips and cookies daily. Four out of 10 adults and more than half of the children reported the consumed fruits daily. Table 2 presents participants’ snack consumption of specific snacks.

### Snack preferences

Children’s five most preferred snacks were potato chips, ice cream, muffins, fruits and corn chips. At the same time, adults’ five most preferred snacks were potato chips, fruits, freshly fried chips, corn chips and cookies (Table 3). More than two-thirds of children indicated they ‘really liked’ ice cream, potato chips, fruits, cakes and chocolates, whereas more than half of adults indicated that they ‘really liked’ fruits, freshly fried chips, ice cream, potato chips, biltong, cookies and chocolates. Table 3 compares the preference for snacks among children and adults. Children indicated they statistically significantly liked potato chips, cheese curls, sweets, chocolates, snack bars, ice cream, cakes and muffins more than adults. In contrast, adults significantly liked savoury biscuits, condensed milk and biltong more than children (Table 2).

**TABLE 3:** Indication of likeness by participants.

Snack	Participants	Really like it (%)	Like it a little (%)	It is okay (%)	Do not like it (%)	Really do not like it (%)	Never tasted it (%)	Mean likeness score	*P*-value
Potato chips	Children	78.4	13.6	6.8	1.1	0	0	4.69	**0.003**
	Adults	59.2	18.4	20.4	2.0	0	0	4.35	
Corn chips	Children	60.9	19.5	12.6	2.3	4.6	0	4.30	0.094
	Adults	49.0	18.4	29.6	3.1	0	0	4.13	
Cheese curls	Children	36.8	24.1	12.6	13.8	12.6	0	3.59	**< 0.001**
	Adults	14.1	15.2	29.3	23.2	15.2	3.0	2.81	
Popcorn	Children	33.3	20.7	24.1	13.8	8.0	0	3.57	0.782
	Adults	26.5	26.5	29.6	10.2	6.1	1.0	3.54	
Peanuts or nuts	Children	33.0	18.2	25.0	11.4	9.1	3.4	3.44	0.093
	Adults	45.4	16.5	19.6	9.3	7.2	2.1	3.77	
Fruit (fresh, canned or dried)	Children	69.3	14.8	9.1	1.1	2.3	3.4	4.38	0.777
	Adults	68.7	10.1	12.1	3.0	4.0	2.0	4.30	
Raisins	Children	23.8	14.3	26.2	14.3	13.1	8.3	2.96	0.651
	Adults	25.8	16.5	22.7	14.4	16.5	4.1	3.08	
Sweets	Children	53.9	19.1	16.9	6.7	2.2	1.1	4.12	**0.006**
	Adults	35.8	15.3	22.4	12.2	8.2	3.1	3.55	
Snack bars	Children	40.2	25.3	19.5	11.5	3.4	-	3.87	**< 0.001**
	Adults	18.8	18.8	34.4	13.5	11.5	3.1	3.10	
Chocolates	Children	67.0	14.8	9.1	3.4	5.7	-	4.34	**0.016**
	Adults	50.5	16.5	16.5	7.2	8.2	1.0	3.91	
Rusk	Children	10.8	16.9	19.3	19.3	13.3	20.5	2.31	0.442
	Adults	12.6	13.7	25.3	13.7	29.5	5.3	2.51	
Cookies	Children	60.5	19.8	10.5	3.5	5.8	-	4.26	0.224
	Adults	51.5	19.2	24.2	1.0	3.0	1.0	4.12	
Biltong or dried wors	Children	22.7	18.2	21.6	11.4	6.8	19.3	2.81	**< 0.001**
	Adults	51.5	19.2	24.2	1.0	3.0	1.0	3.97	
Savoury biscuits	Children	9.5	14.3	19.0	15.5	13.1	28.6	2.06	**0.002**
	Adults	15.6	14.6	35.4	14.6	12.5	7.3	2.84	
Cakes	Children	67.8	18.4	8.0	0	3.4	2.3	4.40	**0.003**
	Adults	50.0	12.2	21.4	6.1	9.2	1.0	3.85	
Tarts	Children	14.5	16.9	25.3	7.2	14.5	21.7	2.45	0.545
	Adults	19.6	13.4	20.6	12.4	21.6	12.4	2.60	
Scones or muffins	Children	60.7	23.8	10.7	2.4	2.4	-	4.38	**0.042**
	Adults	48.0	22.4	23.5	3.1	2.0	1.0	4.08	
Ice cream	Children	80.7	10.2	6.8	2.3	-	-	4.67	**< 0.001**
	Adults	60.6	11.1	12.1	8.1	7.1	1.0	4.07	
Condensed milk	Children	8.3	7.1	17.9	16.7	9.5	40.5	1.67	**0.003**
	Adults	8.4	10.5	29.5	14.7	27.4	9.5	2.29	
Chips (fresh fried)	Children	62.4	21.2	10.6	3.5	2.4	-	4.38	0.965
	Adults	64.9	14.4	12.4	3.1	4.1	1.0	4.30	
High-protein snacks	Children	27.4	23.8	19.0	10.7	9.5	9.5	3.20	0.062
	Adults	34.7	26.3	23.2	8.4	4.2	3.2	3.69	

Note: *P*-value < 0.05 is significant.

### Association between snack consumption frequency and preferences

There was a moderate to strong positive association between the consumption frequency and liking of most snacks, including fruits, snack bars, potato chips and corn chips (Table 4). However, a weak positive relationship between the liking and consumption frequency of cakes, scones or muffins and ice cream among participants was found. Snacks with a strong positive association between consumption frequency and liking by participants were rusks, high-protein snacks and peanuts or nuts.

**TABLE 4:** Relationship between snacks frequently consumed and liked by participants.

Snack	Correlation coefficient (r_s_) between snack consumption frequency and likeness	*p*
Potato chips	0.483	0.0001
Corn chips	0.508	0.0001
Cheese curls	0.570	0.0001
Popcorn	0.568	0.0001
Peanut or nuts	0.624	0.0001
Fruits	0.445	0.0001
Raisins	0.509	0.0001
Sweets	0.537	0.0001
Snack bars	0.570	0.0001
Chocolates	0.550	0.0001
Rusks	0.650	0.0001
Cookies	0.402	0.0001
Biltong or dried wors	0.531	0.0001
Savoury biscuits	0.449	0.0001
Cakes	0.284	0.0001
Tarts	0.606	0.0001
Scones or muffins	0.382	0.0001
Ice cream	0.379	0.0001
Condensed milk	0.539	0.0001
Chips (freshly fried)	0.486	0.0001
High-protein snacks	0.638	0.0001

## Discussion

This study aimed to assess snacking preferences and frequency of children and adults snack consumption to guide the development of nutrient-dense and healthy snacks. This study identified important trends in snack consumption among a community of low SES in South Africa. Our findings showed that more than two-thirds of children consume snacks at least once a day, with nearly half of adults consuming snacks daily.

In our study, the most frequently consumed snacks by survey respondents included fruits, potato chips, corn chips, sweets and cookies. This trend is similar to the reported snack consumption pattern in South Africa. In a snack survey among 200 school children in Grades 4–7, Govender et al. (2018) reported sweets, chips, soft drinks and chocolates are the most consumed snacks. Similarly, in a randomised controlled trial to improve healthy snacks among primary school children in the Western Cape province, Steyn et al. (2015) found that the most consumed snacks by children between the age of 8–10 years were potato chips, sweets and sugar-sweetened beverages. Aside from fruits, the snacks mostly consumed by our study respondents, particularly among children, are nutrient-poor, energy-dense snacks that are either high in added sugar or sodium or low in fibre and micronutrients. Our study findings showed that children significantly consumed more potato chips, corn chips, cheese curls and muffins than adults. The intake of energy-dense, nutrient-poor snacks has been associated with obesity, cardiovascular diseases, non-communicable diseases (NCDs) and the DBM (Barrington & Beresford 2019; Popkin, Adair & Ng 2012; Popkin et al. 2020). In a 5-year Adult Prospective Urban and Rural Epidemiology (PURE) study among South Africans, increased consumption of added sugars was associated with higher waist circumference, body mass index (BMI) and lower HDL-cholesterol levels (Vorster et al. 2014)

When considering the relationship between snacks liked and frequently consumed, a weak positive association between snack consumption frequency and liking was found for ice cream scones or muffins and cakes (Table 4). Fruits and most snacks had a moderate positive association, while peanuts and rusks had a strong positive association. Furthermore, our findings indicate that children and adults in this community may not frequently be consuming the snacks they like. While some snacks were highly preferred, they were not frequently consumed. For instance, 80.7% of children indicated they liked ice cream. However, the mean frequency consumption score of ice cream was lower than 1. Similarly, 67% of children liked cakes, but it had a mean frequency consumption score of 1 (Table 3). Previous research and studies conducted in the communities of the Vaal region in South Africa has consistently indicated a low SES (De Bruyn 2022; Oldewage-Theron et al. 2018; Oldewage-Theron et al. 2014). Studies focusing on this region have revealed significant poverty-related challenges and the need for income-generating activities (De Bruyn 2022; Oldewage-Theron et al. 2018; Oldewage-Theron et al. 2014, 2018). These studies have shown that poverty affects individuals’ and households’ snack and food choices (Du Plessis 2009; Erasmus 2009; Napier, Oldewage-Theron & Makhaye 2018; Oldewage-Theron, Dicks & Napier 2006). The effect of poverty on food choices is evidenced by studies showing that households living in poverty tend to purchase food based on the ability to provide quick satiety rather than nutrient quality when they get relatively more money to spend on food (Govender et al. 2018; Oldewage-Theron et al. 2006). A recent snack sales and consumption survey confirmed increased sales and consumption of cheaper snack brands in 2021 compared to the previous year (IPSOS South Africa 2021).

This study found that fruits are children’s and adult’s most frequently consumed ‘snacks’. This finding is in contrast to several studies that have reported a low intake of fruits and vegetables in South Africa compared to snacks (McHiza et al. 2015; Ronquest-Ross et al. 2015). This could be because this study did not determine the portion sizes of snacks consumed. The study aimed to determine the frequency and predilection of specific snacks. However, it could not account for portion sizes consumed or if a pack or a snack serving was consumed at more than one sitting. Consuming sugary and salty snacks is a cause of public concern in South Africa and is not part of the recommendations for healthy eating. The food-based dietary guidelines for South Africans (FBDG-SA) recommends consuming five servings of fruits and vegetables daily and limiting foods high in added sugars and salt (Vorster, Badham & Venter 2013). While it is applaudable that fruits were highly preferred and frequently consumed by our study respondents, the cost of fruits is generally higher than highly processed snacks and not easily accessible or affordable by low-income individuals and households in South Africa (FAO et al. 2022; Herforth et al. 2020; Temple et al. 2011).

Snacking is often defined differently, as there is no generally accepted definition or classification of snacks. A significant strength of this study is that it defined the term ‘snacks’ and identified major snacks consumed by respondents in each subcategory of children and adults. However, it was not without limitations. The study was conducted within a specific population in a particular region in South Africa. Hence, the snacking preferences and consumption patterns may not be generalisable for all children and adults in South Africa.

## Conclusion

In line with similar studies, this study reported that adults and children in low SES communities in South Africa prefer energy-dense snacks such as ice cream, potato chips, corn chips, muffins and cookies. Similarly, the most frequently consumed snacks are fruits, potato chips, corn chips, cookies and sweets. Apart from fruits, the most popular snacks consumed by the population are high in added sugars, saturated fat and sodium, which are against the South African dietary guidelines for healthy eating. The findings of this study could guide effective nutrition education interventions and programmes on healthy snacking. In addition, the results and findings of this study could be pivotal for snack food producers and manufacturers in developing nutrient-dense snacks that are acceptable to individuals from a low SES community without compromising on taste and affordability. Nutrient-dense snacks low in sugar and sodium should be developed as a substitute for potato chips, corn chips, sweets and cookies. Future research and nutrition education programmes should include teaching households how to prepare nutrient-dense snacks, especially those with little or no added sugar, sodium and low saturated fat content.
